# Nicotine Misuse and Treatment of Schizophrenia Exacerbations in Men: An Observational Study in Poland

**DOI:** 10.3390/ph18091366

**Published:** 2025-09-12

**Authors:** Jakub Grabowski, Leszek Bidzan, Aleksandra Brzozowska

**Affiliations:** 1 Division of Developmental, Psychotic and Geriatric Psychiatry, Department of Psychiatry, Faculty of Medicine, Medical University of Gdańsk, 80-282 Gdańsk, Poland; 2 Department of Health Sciences, Pomeranian University in Słupsk, 76-200 Słupsk, Poland; 3 Adult Psychiatry Scientific Circle, Division of Developmental, Psychotic and Geriatric Psychiatry, Department of Psychiatry, Faculty of Medicine, Medical University of Gdańsk, 80-282 Gdańsk, Poland

**Keywords:** mental health treatment, antipsychotics, psychopharmacology, schizophrenia, psychotic disorders, nicotine misuse

## Abstract

**Background/Objectives**: Prevalence of nicotine misuse among schizophrenia patients is significantly higher than in the general population and is estimated at 70–90%. Past studies have shown that nicotine misuse affects the course of the schizophrenic process in terms of frequency of hospitalizations, age of the first onset, social functioning, and pharmacotherapy, among others. This study aimed to examine associations between smoking and psychopathology, course of hospitalization, doses of administered antipsychotics, and severity of adverse events in men hospitalized for exacerbations of schizophrenia. **Methods**: Protocol procedures were performed in 81 men (40 smokers and 41 non-smokers) and included assessments with a structured interview, laboratory tests, the Positive and Negative Syndrome Scale (PANSS), Montgomery and Asberg Depression Rating Scale (MADRS), Fagerstrom Test for Nicotine Dependence (FTND), and extrapyramidal symptom scales. **Results**: In both groups, a comparable number of patients met the criteria for remission. However, in the pre-discharge period, non-smokers had more severe depressive symptoms measured by MADRS and PANSS than smokers, as well as more severe and more frequent extrapyramidal symptoms. In contrast to previous research, significantly higher doses of antipsychotics measured in chlorpromazine equivalent (CPZE) doses were administered in non-smokers than in smokers (881.1 versus 689.3, *p* = 0.0305). Non-smokers were also more likely to need high doses of medication (>1000 milligrams CPZE) than smokers (43.9% versus 20%, *p* = 0.0212). However, these associations lost statistical significance after adjustment for initial severity and treatment-related factors. Comparison of CPZEs in the context of metabolic pathways suggests that variations in doses are independent of metabolism by cytochrome P450 1A2 (CYP1A2). The results also indicate that nicotine may help to differentiate between negative and depressive symptoms. **Conclusions**: In this male inpatient sample, smokers showed lower depressive symptom scores. Although smoking may affect some symptoms of schizophrenia according to the self-medication hypothesis, therapeutic measures aimed at smoking cessation should not be delayed in this group of patients.

## 1. Introduction

Schizophrenia is a mental disorder characterized by a disintegration of thought processes and emotional reactivity. It affects 1% of the general population in developed countries [1]. It manifests itself by psychopathological abnormalities that were initially divided into two [2] and then into three symptom dimensions [3,4]. Nowadays, in addition to positive (delusions, hallucinations) and negative (passive social withdrawal, monotonous affect, emotional withdrawal) symptoms and disorganization (formal thinking disorder, attention deficit disorder) [5], other symptoms, including affective (depressive), cognitive (impaired working memory and stimulus processing), aggressive (mainly verbal and physical threats, especially when accompanied by alcohol misuse), and pseudo-negative (not directly resulting from schizophrenia, but secondary to other factors, e.g., to positive symptoms, comorbid depression, substance abuse, or social isolation or resulting from adverse drug reactions) are identified [6,7,8,9]. Genetic, environmental, neurobiological, and social factors all seem to play a role in the pathogenesis of the disorder. According to the *Diagnostic and Statistical Manual of Mental Disorders Fourth Edition Text Revision* (DSM-IV-TR), schizophrenia is a disorder that lasts for at least 6 months and includes at least 1 month of active-phase symptoms, i.e., two (or more) of the following: delusions, hallucinations, disorganized speech, grossly disorganized or catatonic behavior, and negative symptoms) [10]. The course of schizophrenia can vary but is generally associated with deterioration of social and occupational functioning, leading in extreme but frequent (10–15%) cases to suicide [11,12].

An equally important issue from a public health perspective is nicotine misuse. Around 25% of the adult population in the European Union smoke cigarettes [13]. There is a similar proportion of people with tobacco addiction worldwide, with 1.1 billion people being current smokers [14]. Each year, more than 8 million people worldwide die from smoking [15]. The risk that a lifelong smoker will die from a nicotine-related disease is 50% [16].

For many years, researchers and clinicians have noted the higher prevalence of nicotine misuse in patients with schizophrenia than in the general population. Among those with schizophrenia, 70–90% are also smokers, depending on the group studied [17,18,19,20,21]. The prevalence of nicotine misuse is much higher not only compared to the general population but also compared to people with other mental disorders (approximately 50% of smokers) [22,23,24]. Patients with schizophrenia are more heavily addicted, smoke more, inhale more heavily, use cigarettes with higher nicotine content, and quit less frequently [25,26,27,28].

Despite initially conflicting findings [29,30], it is now accepted that the risk of schizophrenia is higher among tobacco smokers [31,32,33], although the basis of this relationship is not clear. Reaching for cigarettes may be an early predictor, a symptom of the prodromal phase, or a risk factor for psychosis. The latter hypothesis received strong supporting evidence from a large cohort study of more than 1.5 million people, which indicated that nicotine misuse itself is a factor that prospectively predicts the risk of schizophrenia and that the risk is clearly dose-dependent [34]. Smoking was even shown to be associated with subthreshold psychotic symptoms in a population of healthy young adults (18–35 years) [35]. The interval between smoking initiation and the first psychotic episode (measured in years) was shown to be shorter for schizophrenia than for other psychoses [36]. Patients themselves report that they turn to cigarettes for the same reasons as people without psychotic disorders (for pleasure, relaxation, or due to a sense of physiological dependence) [19], but also to reduce the severity of negative symptoms or adverse effects of medication [37].

Nicotine acts on the central nervous system primarily as an agonist of nicotinic acetylcholinergic receptors (NACh-Rs). NACh-R modulation may improve some symptoms of psychiatric disorders, including schizophrenia. Primarily, improvements in negative symptoms, depressive symptoms, or cognitive impairment (working memory, executive functions, attention) were reported [38]. In addition, cigarette smoking has been associated with lower measured extrapyramidal side effects of antipsychotic drugs (APDs) in some studies [39]. All of the above potentially beneficial effects of tobacco are sometimes collectively referred to as ‘self-medication’ [40].

Nicotine is mainly metabolized by liver enzymes: the 2A6 and 2D6 subunits of cytochrome P450 (CYP2A6, CYP2D6), uridine diphospho(UDP)-glucuronosyltransferase (UGT) 1–4 (UGT1A4), and flavin-containing monooxygenase (FMO). Additionally, it undergoes partial spontaneous metabolism to nornicotine (without enzyme involvement), and approximately 10% of nicotine is excreted unchanged [41,42]. Many of the commonly used APDs interact pharmacokinetically with substances in cigarette smoke. For further consideration, it is worth noting how some of the APDs are metabolized (Table A1). Nicotine alone has a relatively minor effect on the hepatic metabolism of APDs, mainly through the induction of UDP-glucuronyltransferases (UGT1A4, UGT1A9) [43,44], which can lead to lower concentrations of some typical APDs, oxazepam, or propranolol, which is sometimes used to treat APD-induced akathisia. Polycyclic aromatic hydrocarbons (PAHs) play a far greater role. They induce the microsomal hepatic cytochrome P450 enzyme system of CYP1A1, CYP1A2, CYP2A6, CYP2B6, and CYP2E1 [44,45,46]. An inhibitory effect on CYP2D6 receptors is also possible, as smokers have been reported to metabolize nicotine more slowly than non-smokers. Admittedly, CYP2A6 is responsible for 80% of nicotine metabolism, but the effect of PAHs is mainly through its induction, and the inhibition of this enzyme by some components of tobacco smoke is not large enough to explain the phenomenon of slower nicotine metabolism [47].

Smoking patients treated with clozapine have blood levels of the drug reduced by up to 50% compared to non-smokers as a result of the interactions described above, depending on the amount and type of cigarettes smoked [48,49,50,51,52]. This effect is seen even when higher doses of clozapine are used in smokers [53,54], and with concomitant valproate use, drug concentrations may be even lower [55]. This is important because valproic acid or its sodium combination is currently the most commonly added anti-epileptic drug for anticonvulsant prophylaxis in combination with high doses of clozapine. Similar reductions (up to 50%) are also observed in patients using olanzapine [56,57,58]. Cigarette smoking among patients treated with APDs may contribute to the attenuation of some extra-pyramidal symptoms (EPSs) by at least two mechanisms. The PAHs contained in cigarette smoke lower drug concentrations (as described above) and thus lead to reduced dopamine receptor blockade. On the other hand, nicotine, through its direct action on the central nervous system (CNS) (activation of the nigrostriatal part of the dopaminergic system), also reduces the severity of symptoms [59].

This research aimed to examine the associations between cigarette smoking and the psychopathological picture, length of hospitalization, doses and types of medication used, and severity of side effects in patients with schizophrenia. The intention was to verify the hypotheses that smoking affects the course of the disease by alleviating negative and depressive symptoms and reducing the severity of the side effects of antipsychotic treatment. We also hypothesized that smokers require higher doses of antipsychotic drugs than non-smokers. This appears important given the specificities of smoking among patients with schizophrenia described above, the scarcity of scientific data on the impact of smoking on the course of treatment with atypical antipsychotics, and the potential benefits of being able to improve the therapeutic process.

## 2. Results

**Figure 1:**
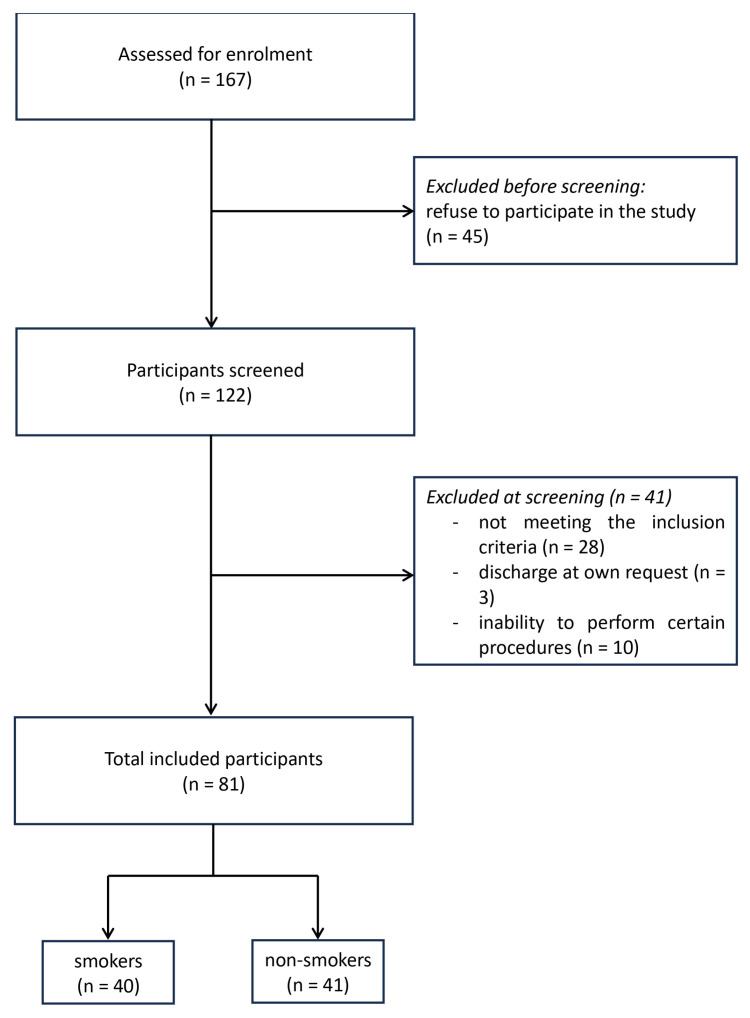
Flowchart of the study participant enrolment process.

A total of 167 men hospitalized for schizophrenia exacerbation were initially qualified for this study, of whom 122 agreed to participate. After excluding those who did not meet the inclusion criteria, those discharged at their own request, and those in whom certain procedures could not be performed, 81 patients (40 smokers and 41 non-smokers) eventually completed the study. Inclusion criteria for this study were as follows: consent to participate in the study, male gender, age 25–55 years, diagnosis of schizophrenia according to DSM-IV-TR, duration of illness for a minimum of 5 years, and hospitalization for an exacerbation of schizophrenia. Reasons for exclusion from the study were as follows: refusal to participate in the study in any period of observation, diagnosis of another Axis I disorder according to DSM-IV-TR, serious somatic illness, especially in the decompensated phase, history of head injury with loss of consciousness or epilepsy, history of neuroinfection, abuse or misuse of psychoactive substances other than nicotine, features of organic CNS damage, nicotine intake by means other than cigarette smoking (gum, patches, pipe smoking, electronic cigarettes, self-rolled cigarettes), and smoking between 1 and 6 cigarettes per day in the past 3 months. Other differential diagnoses were excluded in order to focus exclusively on patients with schizophrenia and the course of their disease. Finally, 81 men were included in the study, of whom 40 smokers and 41 non-smokers. Figure 1 shows a flowchart of the study participant enrolment process.

The criteria for a patient to be classified as a smoker were as follows: a Fagerstrom Test for Nicotine Dependence (FTND) score of 4 or more and smoking at least 7 cigarettes per day in the past 3 months. The following patients were considered non-smokers: those with a FTND score of 0 and those who had not smoked a single cigarette in the past 3 months. This group included both patients who had never smoked and those who had stopped smoking at least 1 year ago. Both groups were homogeneous in terms of demographics, biometric parameters, family history, and concomitant somatic diseases and their treatment (Table 1 and Table A2). Among biometric data, no statistically significant difference in body mass index (BMI) was found between smokers and non-smokers (26.4 vs. 27.3, *p* = 0.26).

Among smokers, the mean age of smoking initiation was 19.2, while the number of cigarettes smoked per day in the past three months ranged from 7 to 60 (mean 22.5). On admission, 20 of the 40 smokers showed features of moderate addiction (FTND = 4–5), and 20 had severe addiction (FTND ≥ 6). During hospitalization, 2 people changed their habits, and at discharge, the proportions were 22 and 18, respectively. Fifteen patients were classified into the subgroup of regular smokers (RS, i.e., 7–19 cigarettes per day) and twenty-five into the subgroup of heavy smokers (HS, i.e., ≥20 cigarettes per day) [60], and this refers only to the 3-month period before admission. None of the patients quit smoking during hospitalization.

### 2.1. Past Course of Disease

Observations in both groups showed no differences regarding the age at first onset (Table 2). Most patients in both groups required hospitalization during the first onset. Smokers, compared to non-smokers, had significantly more disease exacerbations and hospitalizations (12.8 vs. 9.5, *p* = 0.0065; 11.9 vs. 9.1, *p* = 0.0105, respectively). The relationship after dividing into subgroups according to the number of cigarettes smoked and the degree of addiction remained statistically significant only for the group of heavy smokers (Table A3). The percentage of patients discontinuing treatment was very high and similar in both groups. A total of 80% of smokers stopped taking medication at least once without consulting their doctor, or arbitrarily reduced their medication dosage, of which 75% showed such lack of cooperation before their current hospitalization. Among non-smokers, the percentages equaled 78% and 70.7%, respectively. The reported differences between the groups were not statistically significant.

### 2.2. Course of Hospitalization

**Figure 2:**
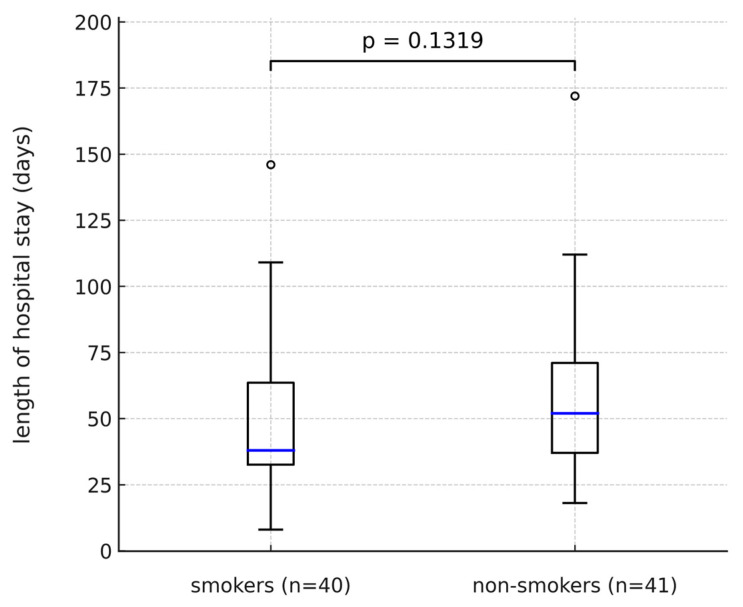
Length of hospital stay in smokers vs. non-smokers.

The average length of treatment was 49.2 days in smokers and it was even lower than in non-smokers (57.6 days), although the results did not meet the criteria for statistical significance (Figure 2).

In order to determine a common denominator for different APDs, the CPZE method was used, according to which the dose of neuroleptic used was converted into milligrams of chlorpromazine. In unadjusted analyses, the mean CPZE administered in smokers was significantly lower than that in non-smokers (689.3 mg vs. 881.1 mg, *p* = 0.0305). The percentage of patients who needed high doses of medication (measured as exceeding 1000 mg CPZE) was also lower in smokers, with the difference reaching significance in the chi-square test (*p* = 0.0212) but not in logistic regression (OR = 0.39, 95% CI [0.14; 1.06], *p* = 0.065) due to methodological differences in small samples (Table 3).

**Figure 3:**
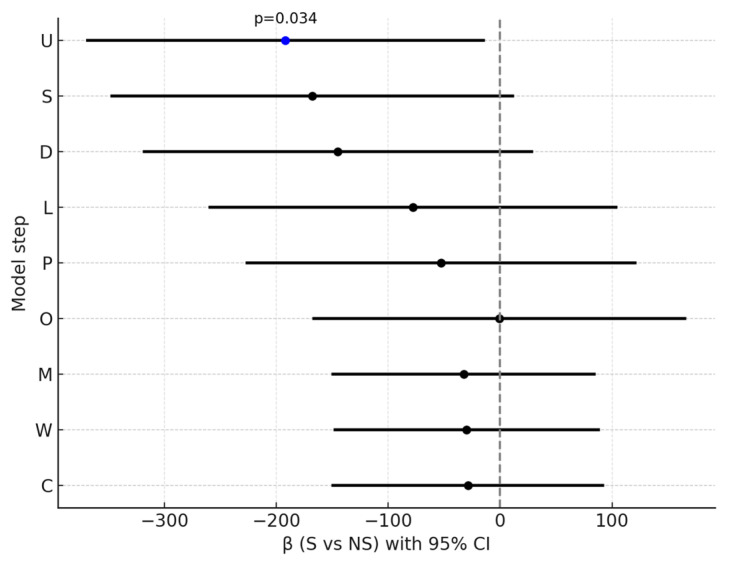
Effect of smoking status (S vs. NS) on CPZE dose across sequential models. β (95% CI) shown for each step: U—unadjusted; S—baseline severity; D—duration of illness; L—LAI use; P—polytherapy; O—olanzapine use; M—CYP1A2 drugs; W—other psychiatric medications; C—caffeine use. Blue dot indicates *p* < 0.05.

**Figure 4:**
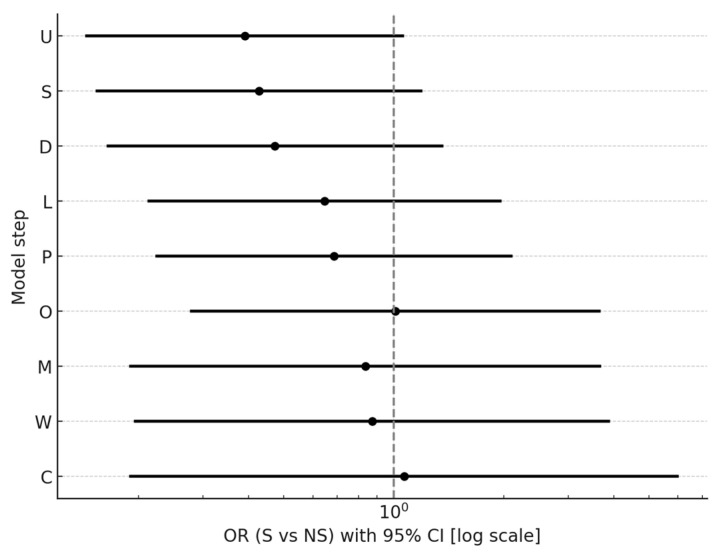
Effect of smoking status (S vs. NS) on the odds of receiving high-dose CPZE (>1000 mg) across sequential models. OR (95% CI) shown for each step: U—unadjusted; S—baseline severity; D—duration of illness; L—LAI use; P—polytherapy; O—olanzapine use; M—CYP1A2 drugs; W—other psychiatric medications; C—caffeine use. Vertical dashed line represents OR = 1.

In stepwise models, the association of smoking with CPZE dose attenuated and lost statistical significance after adjustment for prespecified confounders selected on the basis of a literature review and expert input, including baseline severity (PANSS total and MADRS at admission) and illness duration. Subsequent additions of treatment-related factors (LAI use, antipsychotic polytherapy, olanzapine use, use of antipsychotics predominantly metabolized by CYP1A2, and other psychiatric medications—excluding anticonvulsants) and caffeine use did not significantly change the estimates (Table 4, Figure 3). A similar attenuation pattern was observed for high-dose treatment, with significance lost after adjustment for baseline severity and illness duration and no meaningful change after subsequent adjustments (Table 5, Figure 4). In the fully adjusted model, neither CPZE dose (β = −28.85, 95% CI [−149.37; 91.68], *p* = 0.635) nor high-dose treatment (OR = 0.87, 95% CI [0.20; 3.88], *p* = 0.857) were significantly associated with smoking status.

Patients were most often discharged with the recommendation to take olanzapine and clozapine preparations. Olanzapine was significantly more often administered in smokers. No statistically significant differences were observed for clozapine and the other drugs used (Table A4).

### 2.3. Pharmacotherapy in the Context of CYP1A2 Metabolism

Since the metabolism of both clozapine and olanzapine is primarily mediated by CYP1A2, additional calculations were performed separating drugs by the mode of metabolism. Four groups of patients were identified: patients taking only drugs metabolized mainly by CYP1A2 (M), patients taking only drugs metabolized by routes other than CYP1A2 (NM), patients taking drugs metabolized by CYP1A2 (mainly or partly) or drugs metabolized by CYP1A2 in polytherapy with drugs metabolized by other CYP1A2 pathways (M+), and patients taking drugs metabolized only in part by CYP1A2 or drugs metabolized by CYP1A2 in polytherapy with drugs metabolized by other pathways (NM+). Drugs belonging to the respective metabolic groups are listed in Table A1. There were no statistically significant differences between the M and NM+ groups among either smokers (754.7 mg vs. 575.0 mg, *p* = 0.1690) or non-smokers (855.7 mg vs. 905.4 mg, *p* = 0.7083) (Table A5). When groups M+ and NM were compared, significantly higher CPZEs were observed in M+ groups, both among smokers (562.9 mg vs. 320.3 mg, *p* = 0.0049) and non-smokers (945.0 mg vs. 571.1 mg, *p* = 0.0376) (Table A6). No correlation was detected between cigarette smoking and the group of APDs used (classic, atypical), the type of therapy provided (monotherapy, polytherapy), or the need for additional psychiatric drugs (antidepressants, mood stabilizers, medications that correct drug-induced side effects).

### 2.4. PANSS, MADRS

There were no significant differences between groups in Positive And Negative Symptoms Scale of Schizophrenia (PANSS) total scores at admission and discharge, or in the degree of observed improvement in schizophrenia symptoms (Table A7 and Table A8). A closer analysis of the subscales according to the PANSS-FCTcr model (PANSS consensus five factor) revealed that, at admission, the mean score of the negative factor was significantly lower in smokers than in non-smokers (22.2 vs. 25.5, *p* = 0.0137) (Table A8). After separating the subgroups of heavy smokers, the relationship remained statistically significant only for smokers of smaller amounts of cigarettes. In both groups, a comparable number of patients met the criteria for remission at discharge regardless of the calculation model adopted (PANSS-FCTcr, PANSS central symptoms, PANSS-TScr).

**Figure 5:**
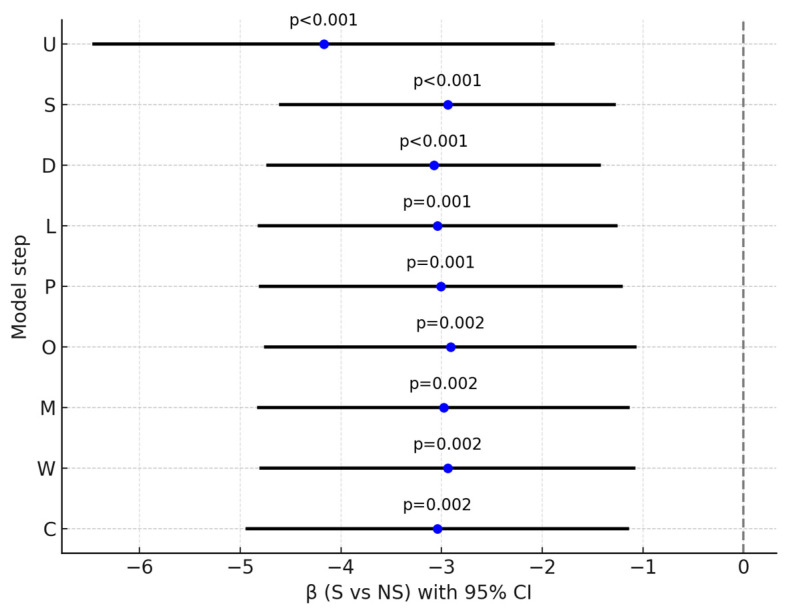
Effect of smoking status (S vs. NS) on MADRS at discharge. β (95% CI) shown for each step: U—unadjusted; S—baseline severity; D—duration of illness; L—LAI use; P—polytherapy; O—olanzapine use; M—CYP1A2 drugs; W—other psychiatric medications; C—caffeine use. Blue dots indicate *p* < 0.05.

There was no statistically significant correlation of Montgomery–Asberg Depression Rating Scale (MADRS) scores at admission relative to the study groups. However, at discharge, MADRS scores were significantly higher in non-smokers (Table A9). This association remained statistically significant after adjustment for baseline severity, illness duration, treatment-related factors, and caffeine use. In the fully adjusted model, smoking remained significantly associated with lower depressive symptom severity at discharge (β = −2.94, 95% CI [−4.79; −1.09], *p* = 0.002) (Table 6, Figure 5).

The observed difference also applied to all separate subgroups among smokers. Specifically, heavy smokers had a β coefficient of −2.97 (95% CI [−5.10; −0.84], *p* = 0.0069), and regular smokers had a β coefficient of −3.16 (95% CI [−5.63; −0.69], *p* = 0.0129) (Table A10). Confirmation of this relationship was revealed by analogous results of the PANSS depression score. Among patients who had marked depressive symptoms at admission (MADRS ≥ 10 points), criteria for remission were significantly more frequently met by smokers (82.9% vs. 39.4%, *p* = 0.0002) (Table 7).

### 2.5. Extrapyramidal Symptom Scales

**Figure 6:**
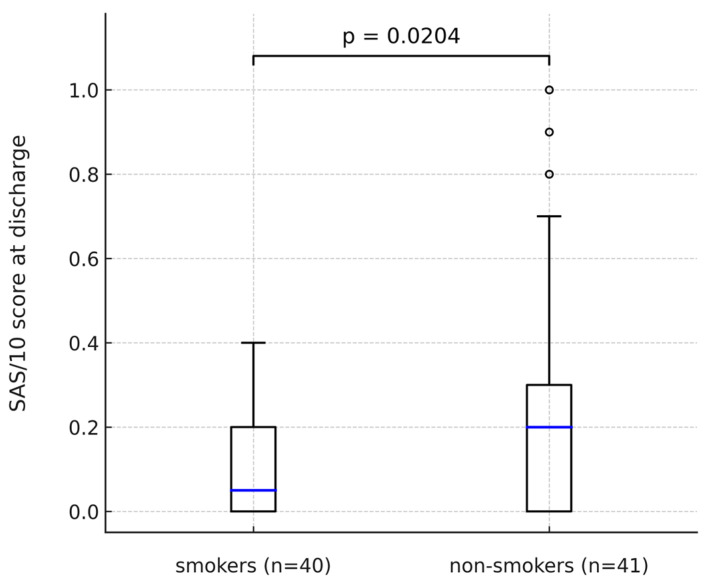
SAS/10 score at discharge in smokers vs. non-smokers.

The Barnes Akathisia Rating Scale (BARS) and Abnormal Involuntary Movement Scale (AIMS) showed no statistically significant differences in relation to cigarette smoking, even after separating the subgroups of heavy smokers those with and severe nicotine addiction. In none of the scales did the scores change significantly at discharge compared to the period of admission. The frequency and severity of parkinsonism as measured by the Simpson–Angus Scale (SAS) at admission in both groups and all subgroups of smokers showed no significant differences. Differences appeared in the evaluation of EPSs in the period before discharge (Figure 6). Parkinsonism was then found significantly more often in non-smokers, regardless of the cutoff point adopted for the SAS: 0.3 or 0.65. It also had a higher severity in this group. SAS scores decreased significantly compared to the admission period only in smokers. There was no statistically significant change in scores in the non-smokers.

### 2.6. Quality of Life

There were no significant differences between smokers and non-smokers in scores for any of the domains, any of the dimensions, or the RAND 36-item Health Survey (SF-36) score. Also, after separating the subgroups by the number of cigarettes smoked and the degree of addiction, almost all scores were very similar. The only difference that met the criterion for statistical significance was in the experience of physical pain in those with severe addiction (FTND ≥ 6) compared to those with moderate addiction (FTND = 4–5).

## 3. Discussion

Given the observational, non-randomized design of this study, all results should be interpreted as associations. The results confirm the strong relationship between smoking and the course of the schizophrenic process described in earlier studies. However, a more detailed analysis of exacerbations, along with their symptomatology and adverse effects of treatment, points to a more complex, multifactorial relationship, which should not be evaluated in isolation from the ongoing pharmacotherapy. Although general observations of the past course of schizophrenia in the two groups showed no differences with regard to the age of the first onset, it should be noted that as many as 27.5% of smokers began smoking during or after the first onset of psychotic symptoms [61,62]. The number of exacerbations and hospitalizations among smokers was also higher [61,63,64,65]. As this relationship remained statistically significant only for the heavy smoker group, there seems to be a relationship between the course of schizophrenia and the number of cigarettes smoked, rather than smoking itself, which in turn indicates that factors other than those determining nicotine use itself may be involved. It is possible that they are merely the result of a more severe dysfunction of neurobiological mechanisms (including the reward system) and a worse course of the disease (increased cigarette smoking could then be linked to attempts to self-medicate some disease symptoms) in heavy smokers.

Preliminary research hypotheses concerning longer periods of hospitalization in smokers as an effect of the need for higher doses of drugs were not confirmed. The most surprising observation was the comparison of the two study groups in terms of the doses of APDs used. Most publications indicate that higher doses of medication are needed in smokers [53,61,66,67,68,69,70], which is in line with popular opinion. The present multivariate regression analyses provide a more nuanced view of these associations. In the unadjusted analysis, smokers received significantly lower CPZE doses than non-smokers and were less likely to be prescribed high-dose treatment (>1000 mg CPZE). After adjustments for baseline psychopathology severity, these associations lost statistical significance, suggesting that the initial differences in doses of APDs may be partly explained by differences in clinical presentation at admission rather than smoking per se. It appears that one of the reasons for obtaining such unexpected results may be the fact that atypical APDs were included in the analysis. The current literature on nicotine misuse in schizophrenia is based on studies conducted on patients taking only or predominantly classical APDs [64,65,68]. Sometimes, patients taking clozapine were also considered [50,53,54]. It is worth noting that even later publications emphasizing higher doses of neuroleptics in smokers sometimes describe studies actually performed in the 1980s and 1990s [65,71], which may make it difficult to assess the problem.

The observations regarding pharmacotherapy in the context of CYP1A2 also seem to indicate that the variation in patients’ CPZE doses is independent of metabolism by CYP1A2. There may be an independent factor influencing the acceleration of CYP1A2-mediated drug metabolism in non-smokers. This requires further research on a larger group and possibly including testing for genetic polymorphisms [72,73,74].

An analysis of the symptomatology of patients in both groups may help to explain the observed differences in medication doses. Smokers and non-smokers did not differ significantly in PANSS scores either at admission or discharge, except for lower negative factor in smokers, as also noted by Krishnadas [75]. The above observations are consistent with results obtained by other researchers regarding the correlation of smoking with negative symptoms in patients hospitalized for an exacerbation of schizophrenia [76], while there was no association with positive symptoms [63]. As negative factor scores were comparable for smokers (including all subgroups) and non-smokers at discharge, it may be that attending physicians, observing the poorer functioning of non-smokers in the domain of negative symptoms, attempted to activate patients with pharmacotherapy, which implied the need for higher doses of APDs. As a result, similar effects to those seen in smokers due to nicotine supply, with regard to psychological state, could be achieved in non-smokers by medication. This would be the other side of the phenomenon Matthews et al. [77] wrote about, indicating that atypical APDs offsetting negative symptoms lead to a reduction in cigarette smoking. At the same time, it is worth noting that the researcher was not responsible for the therapy of any of the patients included in the study. All patients were treated with the normal hospital regimen by different attending physicians who remained unaware of the results of the scales.

The lower severity of negative symptoms could also explain the more frequent use of olanzapine in smokers, without concern for the sedative effects of the drug. Paradoxically, confirmation of this hypothesis may be provided by studies indicating that cigarettes accelerate CYP1A2 metabolism. Their results show that significantly higher doses of clozapine with significantly lower serum levels of clozapine and norclozapine are recorded in smokers [53,54]. Given the substantial health consequences of nicotine misuse and reports identifying cigarette smoking as a major reason for shortening the life span of mentally ill patients by 10–20 years [78], the results suggest that there is no justification for postponing smoking cessation interventions in patients with schizophrenia.

Perceptions of the relationship between cigarette smoking and depression in people with schizophrenia are often contradictory [79,80,81]. In the present study, even though the severity of depressive symptoms on admission did not differ between smokers and non-smokers, at discharge, MADRS scores were significantly lower in smokers and smokers were more likely to reach the criteria for remission.

The study did not reveal a correlation between SAS and MADRS scores, nor between SAS and negativity and depression rates, at either admission or discharge for both study groups. This indicates a low risk of EPSs being mistaken for negative and depressive symptoms, which is particularly important given the higher severity of co-occurring extrapyramidal, depressive, and negative symptoms in non-smokers. The frequency and severity of parkinsonism as measured by the SAS showed no significant differences between smokers and non-smokers at admission. However, prior to discharge, parkinsonism was found significantly more often in non-smokers. Given that the majority of patients were not taking medication prior to their current hospital stay, it can be assumed that the EPSs appearing on admission were similar in both groups due to the intensive antipsychotic treatment introduced, and that with the time of hospitalization, between-group differences in EPSs were observed, with lower EPSs in smokers, as described by many researchers [61,82,83]. On the other hand, the greater severity of EPSs in non-smokers may be responsible for the higher doses of APDs used observed in this study. This is supported by the correlation found between SAS scores at discharge and the dose of CPZE used at that time for both smokers and non-smokers.

Most studies to date have indicated a significantly poorer quality of life in patients with schizophrenia who smoke cigarettes compared to non-smokers [84,85]. This is primarily related to the numerous somatic diseases associated with nicotine misuse. The present study did not reveal a relationship between smoking and the presence of comorbidities, frequency of medication use for somatic conditions, or deviations in basic laboratory tests and biometric measurements. Similar results of the above quality-of-life parameters have also been reported in the past [86]. This is reflected by the patients’ self-assessment via the SF-36 questionnaire. The significant differences in experiencing physical pain may be related to the well-known but still unexplained phenomenon of a higher pain threshold in patients who smoke or have schizophrenia in general [87,88].

The biggest limitation of the study appears to be the relatively small size of the study groups, which leads to reduced statistical power and prevents extrapolation of the results to the general population. Another serious limitation was the categorization into smokers and non-smokers based only on the patients’ account (without objectification, e.g., by measuring serum cotinine levels or carbon monoxide content in exhaled air). The number of cigarettes smoked by the patient in the last three months was only assessed on admission, and assessment at discharge was only indirect and imprecise, based on one of the FTND items. During hospitalization, misuse may have decreased in some patients, potentially affecting drug metabolization. However, changes in metabolic induction usually appear after several weeks, and for a full CYP1A2 induction, smoking only 7–12 cigarettes per day is sufficient [46].

It should also be noted that the consumption of coffee, whose metabolism is highly dependent on CYP1A2 activity [89], was not recorded quantitatively, but only in binary terms. Smokers need up to four times the dose of caffeine to achieve the same plasma concentration of this substance. Caffeine itself, in turn, can increase the concentration of clozapine and olanzapine by neutralizing or reducing the effect of cigarettes on CYP1A2, thus reducing the need for higher doses of medication [90].

The study also did not take into account the genetic polymorphism of CYP1A2. It may affect drug metabolism and, consequently, the doses used, but previous studies on stable outpatient groups have not revealed any clinically relevant differences in this regard between smokers and non-smokers [50]. CYP2A6 and CYP2D6 with their polymorphisms were also not considered, although they may be related to the development of nicotine misuse and the rate of nicotine metabolism [44,91].

It should be strongly emphasized that the findings cannot be generalized to women. Due to significant gender differences regarding the course and treatment of schizophrenia and the specific nature of nicotine misuse, only men were eligible to participate in the study. In men, the first symptoms of schizophrenia appear earlier, their pre-onset functioning is worse, and they present more negative symptoms and cognitive deficits. Women are more likely to experience affective symptoms, persecutory delusions, and auditory hallucinations while responding faster and better to antipsychotic drugs [92,93]. Women are at increased risk of extrapyramidal side effects, including akathisia, and the course of tardive dyskinesia is more severe [66,94]. The pharmacokinetics of psychotropic drugs also differ between the sexes, which may lead to pharmacodynamic changes. The most frequently highlighted variables include the rate and amount of drug absorbed, volume of distribution, renal clearance and elimination of the substance, and its hepatic metabolism. This translates directly into the need for lower doses of some drugs in women [95,96]. It has also been proven that the rate of nicotine and cotinine metabolism differs between the sexes and is significantly faster in women [97,98].

Another limitation of the study was the lack of comprehensive tests aimed at excluding secondary schizophrenia (i.e., substance use and general conditions). Such tests might include magnetic resonance imaging (MRI), electroencephalography (EEG), lumbar puncture, or drug urinalysis. These would have verified the information obtained from the subjective and objective examinations and the existing medical records and confirmed that all patients included in the study meet criterion E for the diagnosis of schizophrenia according to DSM-IV-TR.

This study originally did not include cognitive endpoints. Cognitive testing would likely have been confounded by early inpatient factors (e.g., sedation, acute symptoms, sleep disturbances) and differences in pharmacological treatment between patients. Atypical APDs appear to have beneficial effects on other domains of cognitive functioning. It is also suggested that the beneficial effect of smoking on cognitive function is likely to be temporary. The dynamics of this phenomenon may vary from patient to patient, which further complicates the analysis. To date, studies on the impact of smoking on cognitive function have not yielded conclusive results. There is a clear need for extensive research focusing on this specific issue.

Compliance with medication recommendations was not verified objectively. The use of drug level or pK measurements could provide a better level of evidence. Other potential confounding factors include acute stress at admission and timing of assessments, duration of illness, change in smoking intensity during hospitalization, dietary CYP1A2 modulators, and substance abuse history (other than nicotine).

## 4. Materials and Methods

The study was conducted at the Regional Psychiatric Hospital in Gdansk between 2012 and 2016. The number of men initially qualified for the study (167) corresponds to the number of men who were hospitalized at that time for an exacerbation of schizophrenia. The study plan included examining each patient twice during hospitalization. The first examination took place on admission or within three days of the patient’s admission to the hospital ward and included a complete clinical examination, measurement of vital signs, laboratory tests (complete blood count), erythrocyte sedimentation rate (ESR), glucose, lipid profile, urinalysis), and assessment of mental and neurological status. The following tools were used: PANSS, MADRS, assessment of adverse symptoms of treatment (SAS, BARS, AIMS), and FTND. In addition, a structured interview describing the previous course of the disease was conducted. The second examination took place in the period prior to discharge (up to 7 days before discharge) and included an assessment of mental and neurological status, examination with PANSS, MADRS, SAS, BARS, AIMS, SF-36, and FTND, and a supplementary structured interview. The study was conducted according to the guidelines of the Declaration of Helsinki and approved by the Institutional Ethics Committee of the Medical University of Gdańsk (NKEBN/257/2011, decision of 12 July 2011).

Statistical calculations were performed using the statistical package StatSoft Incorporated (2014) Statistica version 12.0 and an Excel spreadsheet. The significance of the differences between two groups was tested using Student’s *t*-test (or, in the absence of homogeneity of variances, Welch’s test) or the Mann–Whitney U. The significance of differences between more than two groups was checked by analysis of variance (ANOVA) or the Kruskal–Wallis test. Chi-square tests of independence were used for qualitative variables. In all calculations, the significance level was taken as *p* = 0.05.

To examine the influence of potential confounders, stepwise hierarchical regression analyses were conducted. Potential confounding variables were identified a priori based on a literature review and expert input. Linear regression was applied to continuous and logistic regression to the binary outcome of high-dose treatment. OpenAI ChatGPT (version 5) was used to prepare the figures and regression calculations.

Inclusion and exclusion criteria were clearly defined to ensure a consistent study population and reduce selection bias. Data collection procedures were standardized across all participants, and where possible, data were obtained from reliable records rather than self-report to limit recall bias.

Drug doses were converted to CPZE based on scientific sources. The original studies included guidelines for converting doses only for first-generation APDs [99,100]. Using more recent sources, equivalent doses were established for atypical drugs [101], including risperidone long-acting injectable (LAI) [102] and olanzapine LAI [103,104], as well as for first-generation extended-release haloperidol (based on a bioavailability study) [105].

## 5. Conclusions

The study is another insight into the problem of nicotine misuse in schizophrenia. It seems to present a new perspective, as it does not confirm some of the previous concepts regarding the role of cigarette smoking in this disorder. The results suggest the need to address issues such as the overlap between negative and depressive symptoms in clinical practice and the unclear association between smoking status and EPSs observed at discharge (i.e., at a later stage of antipsychotic therapy) rather than at admission. The main problem in establishing the real role of nicotine in the treatment of schizophrenia seems to be the lack of high-quality randomized trials. There is also still a lack of multidisciplinary studies in this field, where genetic, biochemical, neurophysiological, psychological factors, and the broader social context would be taken into account, in addition to patient functioning in terms of psychopathology (along with cognitive functioning, which was omitted from this study) and drug side effects. After all, cigarette smoking, as a phenomenon, is part of a more complex picture and is only an element of the functioning of patients with schizophrenia. The results should therefore not be taken as a foundation for determining causal relationships but rather as an incentive to revisit earlier publications whose theses have become a fixed part of our understanding of the relationship between smoking and the course of the schizophrenic process. Further multicentric studies with higher sample sizes are needed in the future to corroborate the results of this study. Given the gender differences discussed above, similar studies should be conducted among women.

## Figures and Tables

**Table 1:** Demographic characteristics of smokers and non-smokers.

	S (n = 40)	NS (n = 41)	Overall (n = 81)	*p*
Marital status				0.5163
single	35 (87.5%)	32 (78.0%)	67 (82.7%)	
divorced	2 (5.0%)	3 (7.3%)	5 (6.2%)	
married	3 (7.5%)	6 (14.6%)	9 (11.1%)	
Education				0.7148
primary school	10 (25.0%)	7 (17.1%)	17 (21.0%)	
vocational training	14 (35.0%)	13 (31.7%)	27 (33.3%)	
secondary school	13 (32.5%)	18 (43.9%)	31 (38.3%)	
higher education	3 (7.5%)	3 (7.3%)	6 (7.4%)	
Residence				0.6270
countryside	2 (5.0%)	5 (12.2%)	7 (8.6%)	
city with <50 k inhabitants	8 (20.0%)	7 (17.1%)	15 (18.5%)	
city with 50–100 k inhabitants	1 (2.5%)	2 (4.9%)	3 (3.7%)	
city with >100 k inhabitants	29 (72.5%)	27 (65.9%)	56 (69.1%)	
Home environment				0.6361
living alone	6 (15.0%)	5 (12.2%)	11 (13.6%)	
living with family of origin	28 (70.0%)	30 (73.2%)	58 (71.6%)	
living with family of procreation	4 (10.0%)	6 (14.6%)	10 (12.3%)	
other (living with housemates)	1 (2.5%)	0 (0.0%)	1 (1.2%)	
other (supported housing)	1 (2.5%)	0 (0.0%)	1 (1.2%)	
Family of origin				0.5825
incomplete	20 (50.0%)	18 (43.9%)	38 (46.9%)	
complete	20 (50.0%)	23 (56.1%)	43 (53.1%)	
Criminal record				0.3052
convicted	31 (77.5%)	35 (85.4%)	66 (81.5%)	
never convicted	9 (22.5%)	5 (12.2%)	14 (17.3%)	
proceeding in progress	0 (0.0%)	1 (2.4%)	1 (1.2%)	

S—smokers; NS—non-smokers.

**Table 2:** Comparative characteristics—past course of disease.

	S (n = 40)	NS (n = 41)	Overall (n = 81)	*p*
Age at first onset				0.4584
mean (SD)	22.8 (5.0)	24.3 (6.6)	23.5 (5.9)	
range	16.0–40.0	13.0–45.0	13.0–45.0	
median	23.0	23.0	23.0	
95% CI	[21.2; 24.3]	[22.2; 26.4]	[22.2; 24.8]	
Number of exacerbations				**0.0065**
mean (SD)	12.8 (9.2)	9.5 (12.8)	11.1 (11.2)	
range	1.0–42.0	1.0–70.0	1.0–70.0	
median	13.0	6.0	8.0	
95% CI	[9.8; 15.7]	[5.5; 13.6]	[8.7; 13.6]	
Number of hospitalizations				**0.0105**
mean (SD)	11.9 (9.1)	9.1 (12.8)	10.5 (11.2)	
range	1.0–38.0	1.0–70.0	1.0–70.0	
median	10.5	5.0	7.0	
95% CI	[9.0; 14.8]	[5.1; 13.1]	[8.0; 13.0]	

S—smokers; NS—non-smokers; SD—standard deviation; CI—confidence interval; bold values indicate statistical significance.

**Table 3:** Comparative characteristics in terms of drug doses used in CPZE (unadjusted).

	S (n = 40)	NS (n = 41)	Overall (n = 81)	*p*
CPZE				**0.0305**
mean (SD)	689.3 (379.7)	881.1 (418.2)	786.4 (408.7)	
range	100.0–1800.0	200.0–1600.0	100.0–1800.0	
median	600.0	900.0	680.0	
95% CI	[567.9; 810.7]	[749.2; 1013.1]	[696.0; 876.8]	
CPZE high dose				**0.0212**
n (%)	8 (20%)	18 (43.9%)	26 (32.1%)	

S—smokers; NS—non-smokers; SD—standard deviation; CI—confidence interval; bold values indicate statistical significance.

**Table 4:** Sequential adjustment of the association between smoking status and CPZE dose.

Step	Model	β (S vs. NS)	95% CI	*p*	R^2^
U	unadjusted	−191.85	[−368.62; −15.07]	**0.034**	0.056
S	+baseline severity	−167.72	[−346.72; 11.28]	0.066	0.091
D	+duration of illness	−144.95	[−318.26; 28.35]	0.100	0.168
L	+LAI	−77.96	[−259.38; 103.46]	0.395	0.213
P	+polytherapy	−52.77	[−226.04; 120.51]	0.546	0.299
O	+olanzapine	−0.46	[−166.23; 165.31]	0.996	0.390
M	+CYP1A2 drugs	−32.56	[−149.30; 84.17]	0.580	0.703
W	+other psychiatric medications	−29.91	[−147.73; 87.92]	0.614	0.704
C	+caffeine use	−28.85	[−149.37; 91.68]	0.635	0.704

S—smokers; NS—non-smokers; β—estimated parameter of the regression model; CI—confidence interval; R^2^—coefficient of determination; bold value indicates statistical significance.

**Table 5:** Sequential adjustment of the association between smoking status and high CPZE dose.

Step	Model	OR	95% CI	*p*	Pseudo R^2^
U	unadjusted	0.39	[0.14; 1.06]	0.065	0.036
S	+baseline severity	0.43	[0.15; 1.19]	0.103	0.057
D	+duration of illness	0.47	[0.16; 1.35]	0.162	0.095
L	+LAI	0.65	[0.21; 1.95]	0.438	0.125
P	+polytherapy	0.69	[0.22; 2.10]	0.507	0.137
O	+olanzapine	1.01	[0.28; 3.65]	0.988	0.286
M	+CYP1A2 drugs	0.84	[0.19; 3.66]	0.811	0.421
W	+other psychiatric medications	0.87	[0.20; 3.88]	0.857	0.444
C	+caffeine use	1.07	[0.19; 5.99]	0.941	0.447

OR—odds ratio; CI—confidence interval; pseudo-R^2^—coefficient of determination.

**Table 6:** Sequential adjustment of the association between smoking status and MADRS score at discharge.

Step	Model	β (S vs. NS)	95% CI	*p*	R^2^
U	unadjusted	−4.17	[−6.45; −1.89]	**<0.001**	0.144
S	+baseline severity	−2.94	[−4.60; −1.28]	**<0.001**	0.576
D	+duration of illness	−3.08	[−4.72; −1.43]	**<0.001**	0.591
L	+LAI	−3.04	[−4.81; −1.27]	**0.001**	0.591
P	+polytherapy	−3.01	[−4.80; −1.22]	**0.001**	0.592
O	+olanzapine	−2.91	[−4.75; −1.08]	**0.002**	0.593
M	+CYP1A2 drugs	−2.98	[−4.81; −1.15]	**0.002**	0.601
W	+other psychiatric medications	−2.94	[−4.79; −1.09]	**0.002**	0.602
C	+caffeine use	−3.04	[−4.93; −1.15]	**0.002**	0.605

S—smokers; NS—non-smokers; β—estimated parameter of the regression model; CI—confidence interval; R^2^—coefficient of determination; bold values indicate statistical significance.

**Table 7:** Comparative characteristics in terms of remission achieved during treatment for depressive symptoms at discharge (MADRS < 10) in those with depressive symptoms at admission (MADRS ≥ 10).

	S (n = 40)	NS (n = 41)	*p*
remission in depression	29 (82.9%)	13 (39.4%)	**0.0002**

S—smokers; NS—non-smokers; bold value indicates statistical significance.

## Data Availability

The data presented in this study are available on request from the corresponding author due to privacy reasons.

## Appendix A

**Table A1:** Categorization of APDs taken by patients in the study by their metabolism [106,107,108].

Drugs Metabolized Mainly by CYP1A2	Drugs Metabolized Partlyby CYP1A2	Drugs Metabolized by Pathways Other than CYP1A2
clozapine olanzapine	haloperidol levomepromazine perazine	amisulpride aripiprazole chlorprothixene flupentixol quetiapine risperidone zuclopenthixol

**Table A2:** Baseline characteristics in terms of interview data.

	S (n = 40)	NS (n = 41)	Overall (n = 81)	*p*
mental disorders in family	18 (45.0%)	17 (41.5%)	35 (43.2%)	0.7480
alcoholism in family	15 (37.5%)	20 (48.8%)	35 (43.2%)	0.3055
domestic violence	14 (35.0%)	17 (41.5%)	31 (38.3%)	0.5496
additional somatic treatment	13 (32.5%)	22 (53.7%)	35 (43.2%)	0.0546
hyperlipidaemia	8 (20.0%)	10 (24.4%)	18 (22.2%)	0.6347
metabolic syndrome	5 (12.5%)	9 (22.0%)	14 (17.3%)	0.2607
arterial hypertension	4 (10.0%)	11 (26.8%)	15 (18.5%)	0.0512

S—smokers, NS—non-smokers.

**Table A3:** Comparative characteristics—past course of disease with subgrouping into regular smokers and heavy smokers.

	RS (n = 15)	HS (n = 25)	NS (n = 41)	*p*
Age of first onset				0.5339
mean (SD)	22.5 (6.0)	22.9 (4.5)	24.3 (6.6)	
range	16.0–40.0	16.0–31.0	13.0–45.0	
median	21.0	23.0	23.0	
95% CI	[19.2; 25.8]	[21.0; 24.7]	[22.2; 26.4]	
Number of hospitalizations				**0.0171**
mean (SD)	9.9 (8.5)	13.1 (9.4)	9.1 (12.8)	
range	1.0–30.0	2.0–38.0	1.0–70.0	
median	8.0	11.01	5.01	
95% CI	[5.3; 14.6]	[9.2; 17.0]	[5.1; 13.1]	

RS—regular smokers, HS—heavy smokers, NS—non-smokers, SD—standard deviation, CI—confidence interval, bold value indicates statistical significance.

**Table A4:** Comparative characteristics in terms of types of antipsychotic medication prescribed at discharge.

	S (n = 40)	NS (n = 41)	Overall (n = 81)	*p*
olanzapine	23 (57.5%)	13 (31.7%)	36 (44.4%)	**0.0195**
clozapine	10 (25.0%)	18 (43.9%)	28 (34.6%)	0.0737
risperidone	9 (22.5%)	4 (9.8%)	13 (16.0%)	0.1182
chlorprothixene	3 (7.5%)	1 (2.4%)	4 (4.9%)	0.2932
zuclopenthixol	9 (22.5%)	8 (19.5%)	17 (21.0%)	0.7413
quetiapine	1 (2.5%)	5 (12.2%)	6 (7.4%)	0.0958
aripiprazole	4 (10.0%)	8 (19.5%)	12 (14.8%)	0.2283
levomepromazine	3 (7.5%)	2 (4.9%)	5 (6.2%)	0.6240
haloperidol	2 (5.0%)	1 (2.4%)	3 (3.7%)	0.5417
flupenthixol	0 (0.0%)	3 (7.3%)	3 (3.7%)	0.0813
other	0 (0.0%)	3 (7.3%)	3 (3.7%)	0.0813

S—smokers, NS—non-smokers, other—amisulpride, perazine, bold value indicates statistical significance.

**Table A5:** Comparative characteristics in terms of CPZE doses in smokers and non-smokers by subgroups of patients taking only drugs metabolized mainly by CYP1A2 and other patients.

		NM+ (n = 14)	M (n = 26)	Overall (n = 40)	*p*
S					0.1690
	mean (SD)	575.0 (357.2)	754.7 (380.0)	691.8 (377.7)	
	range	100.0–1100.0	400.0–1800.0	100.0–1800.0	
	median	550.0	600.0	600.0	
	95% CI	[368.8; 781.2]	[601.2; 908.2]	[571.0; 812.6]	
		**NM+ (n = 21)**	**M (n = 20)**	**Overall (n = 41)**	***p***
NS					0.7083
	mean (SD)	905.4 (468.1)	855.7 (369.1)	881.1 (418.2)	
	range	200.0–1600.0	200.0–1440.0	200.0–1600.0	
	median	900.0	875.0	900.0	
	95% CI	[692.4; 1118.5]	[682.9; 1028.4]	[749.2; 1013.1]	

S—smokers, NS—non-smokers, M—patients taking only drugs metabolized mainly by CYP1A2, NM+—other patients, SD—standard deviation, CI—confidence interval.

**Table A6:** Comparative characteristics in terms of CPZE doses in smokers and non-smokers by subgroups of patients taking drugs metabolized with and without CYP1A2 involvement.

		NM (n = 7)	M+ (n = 33)	Overall (n = 40)	*p*
S					0.0049
	mean (SD)	320.3 (228.4)	562.9 (210.3)	520.5 (230.2)	
	range	100.0–775.0	353.0–1354.0	100.0–1354.0	
	median	250.0	536.0	474.0	
	95% CI	[109.1; 531.5]	[488.4; 637.5]	[446.8; 594.1]	
		**NM (n = 7)**	**M+ (n = 34)**	**Overall (n = 41)**	***p***
NS					0.0376
	mean (SD)	571.1 (335.1)	945.0 (408.6)	881.1 (418.2)	
	range	200.0–1141.0	200.0–1600.0	200.0–1600.0	
	median	650.0	950.0	900.0	
	95% CI	[261.3; 881.0]	[802.4; 1087.5]	[749.2; 1013.1]	

S—smokers, NS—non-smokers, M+—patients taking drugs with CYP1A2, NM—patients taking drugs metabolized without CYP1A2 involvement, SD—standard deviation, CI—confidence interval.

**Table A7:** Comparative characteristics in terms of PANSS at admission.

	S (n = 40)	NS (n = 41)	Overall (n = 81)	*p*
PANNS overall				0.2901
mean (SD)	115.0 (17.0)	118.0 (15.9)	116.5 (16.4)	
range	80.0–149.0	75.0–150.0	75.0–150.0	
median	115.0	118.0	117.0	
95% CI	[109.6; 120.4]	[112.9; 123.0]	[112.9; 120.1]	
General psychopathology				0.1497
mean (SD)	33.0 (6.2)	34.8 (6.0)	33.9 (6.1)	
range	20.0–46.0	22.0–46.0	20.0–46.0	
median	32.5	36.0	34.0	
95% CI	[31.0; 35.0]	[32.9; 36.7]	[32.6; 35.3]	
Positive symptoms				0.9322
mean (SD)	16.5 (4.1)	16.4 (4.2)	16.5 (4.1)	
range	9.0–25.0	7.0–23.0	7.0–25.0	
median	16.0	17.0	16.0	
95% CI	[15.2; 17.8]	[15.1; 17.7]	[15.5; 17.4]	
Negative symptoms				**0.0137**
mean (SD)	22.2 (4.8)	25.5 (5.4)	23.9 (5.3)	
range	13.0–30.0	17.0–36.0	13.0–36.0	
median	22.0	25.0	24.0	
95% CI	[20.7; 23.7]	[23.8; 27.2]	[22.7; 25.1]	
Dizorganization coefficient				0.6001
mean (SD)	13.5 (3.1)	13.1 (3.4)	13.3 (3.2)	
range	8.0–20.0	6.0–21.0	6.0–21.0	
median	13.0	13.0	13.0	
95% CI	[12.5; 14.5]	[12.0; 14.2]	[12.6; 14.0]	
Activity coefficient				0.5115
mean (SD)	17.1 (6.9)	16.2 (6.3)	16.6 (6.6)	
range	6.0–26.0	4.0–26.0	4.0–26.0	
median	18.5	16.0	17.0	
95% CI	[14.8; 19.3]	[14.2; 18.2]	[15.2; 18.1]	
Affective coefficient				0.2944
mean (SD)	7.9 (3.8)	8.6 (3.6)	8.3 (3.7)	
range	3.0–15.0	3.0–18.0	3.0–18.0	
median	7.0	8.0	7.0	
95% CI	[6.7; 9.1]	[7.5; 9.8]	[7.5; 9.1]	

S—smokers, NS—non-smokers, SD—standard deviation, CI—confidence interval, bold value indicates statistical significance.

**Table A8:** Comparative characteristics in terms of PANSS at discharge.

	S (n = 40)	NS (n = 41)	Overall (n = 81)	*p*
PANNS overall				0.2879
mean (SD)	70.0 (13.2)	74.0 (16.6)	72.0 (15.1)	
range	49.0–108.0	42.0–109.0	42.0–109.0	
median	68.5	71.0	69.0	
95% CI	[65.8; 74.2]	[68.7; 79.2]	[68.7; 75.3]	
General psychopathology				0.6266
mean (SD)	20.0 (4.8)	21.1 (6.4)	20.6 (5.7)	
range	11.0–30.0	11.0–38.0	11.0–38.0	
median	20.0	20.0	20.0	
95% CI	[18.4; 21.5]	[19.1; 23.2]	[19.3; 21.8]	
Positive symptoms				0.6001
mean (SD)	7.2 (2.6)	7.7 (3.2)	7.4 (2.9)	
range	4.0–13.0	4.0–15.0	4.0–15.0	
median	6.0	7.0	7.0	
95% CI	[6.3; 8.0]	[6.7; 8.7]	[6.8; 8.1]	
Negative symptoms				0.6199
mean (SD)	18.8 (4.9)	19.8 (6.4)	19.3 (5.7)	
range	10.0–30.0	9.0–34.0	9.0–34.0	
median	18.0	19.0	18.0	
95% CI	[17.2; 20.3]	[17.7; 21.8]	[18.0; 20.5]	
Dizorganization coefficient				0.4414
mean (SD)	9.4 (2.6)	9.2 (3.4)	9.3 (3.0)	
range	5.0–16.0	3.0–17.0	3.0–17.0	
median	9.0	8.0	9.0	
95% CI	[8.6; 10.2]	[8.1; 10.3]	[8.6; 10.0]	
Activity coefficient				0.4498
mean (SD)	6.3 (1.9)	6.6 (2.0)	6.4 (2.0)	
range	4.0–12.0	4.0–15.0	4.0–15.0	
median	6.0	6.0	6.0	
95% CI	[5.7; 6.9]	[5.9; 7.2]	[6.0; 6.9]	
Affective coefficient				**0.0258**
mean (SD)	4.6 (1.8)	5.4 (1.9)	5.0 (1.9)	
range	3.0–11.0	3.0–13.0	3.0–13.0	
median	4.0	5.0	5.0	
95% CI	[4.0; 5.1]	[4.8; 6.0]	[4.6; 5.4]	

S—smokers, NS—non-smokers, SD—standard deviation, CI—confidence interval, bold value indicates statistical significance.

**Table A9:** Comparative characteristics in terms of the MADRS.

	S (n = 40)	NS (n = 41)	Overall (n = 81)	*p*
MADRS admission				0.2434
mean (SD)	18.7 (10.3)	22.3 (12.4)	20.5 (11.5)	
range	0.0–47.0	2.0–48.0	0.0–48.0	
median	16.5	19.0	18.0	
95% CI	[15.4; 22.0]	[18.4; 26.2]	[18.0; 23.0]	
MADRS discharge				**0.0005**
mean (SD)	6.0 (4.6)	10.1 (5.7)	8.1 (5.5)	
range	0.0–16.0	0.0–26.0	0.0–26.0	
median	5.5	10.0	7.0	
95% CI	[4.5; 7.4]	[8.4; 11.9]	[6.9; 9.3]	

S—smokers, NS—non-smokers, SD—standard deviation, CI—confidence interval, bold value indicates statistical significance.

**Table A10:** Association between smoking category (FTND) and MADRS score at discharge adjusted for confounding factors (reference category: NS).

Predictor	β	95% CI	*p*
HS status	−2.97	[−5.10; −0.84]	**0.0069**
RS status	−3.16	[−5.63; −0.69]	**0.0129**

HS—heavy smokers, RS—regular smokers, NS—non-smokers, β—estimated parameter of the regression model, CI—confidence interval, bold values indicate statistical significance.
